# Unequivocal Identification of Subpopulations in Putative Multiclonal *Trypanosoma cruzi* Strains by FACs Single Cell Sorting and Genotyping

**DOI:** 10.1371/journal.pntd.0001722

**Published:** 2012-07-10

**Authors:** Helder Magno Silva Valadares, Juliana Ramos Pimenta, Marcela Segatto, Vanja Maria Veloso, Mônica Lúcia Gomes, Egler Chiari, Kenneth John Gollob, Maria Terezinha Bahia, Marta de Lana, Glória Regina Franco, Carlos Renato Machado, Sérgio Danilo Junho Pena, Andréa Mara Macedo

**Affiliations:** 1 Departamento de Bioquímica e Imunologia, Instituto de Ciências Biológicas, Universidade Federal de Minas Gerais, Belo Horizonte, Minas Gerais, Brazil; 2 Universidade Federal de São João del-Rey, Campus Centro-Oeste Dona Lindu, Divinópolis, Minas Gerais, Brazil; 3 Departamento de Análises Clínicas, Escola de Farmácia, Universidade Federal de Ouro Preto, Ouro Preto, Minas Gerais, Brazil; 4 Departamento de Ciências Básicas da Saúde, Universidade Estadual de Maringá, Maringá, Paraná, Brazil; 5 Departamento de Parasitologia, Instituto de Ciências Biológicas, Universidade Federal de Minas Gerais, Belo Horizonte, Minas Gerais, Brazil; 6 Instituto de Ensino e Pesquisa da Santa Casa de Belo Horizonte, Belo Horizonte, Minas Gerais, Brazil; 7 Departamento de Ciências Biológicas, Instituto de Ciências Exatas e Biológicas, Universidade Federal de Ouro Preto, Ouro Preto, Minas Gerais, Brazil; University of Pittsburgh, United States of America

## Abstract

*Trypanosoma cruzi*, the etiological agent of Chagas disease, is a polymorphic species. Evidence suggests that the majority of the *T. cruzi* populations isolated from afflicted humans, reservoir animals, or vectors are multiclonal. However, the extent and the complexity of multiclonality remain to be established, since aneuploidy cannot be excluded and current conventional cloning methods cannot identify all the representative clones in an infection. To answer this question, we adapted a methodology originally described for analyzing single spermatozoids, to isolate and study single *T. cruzi* parasites. Accordingly, the cloning apparatus of a Fluorescence-Activated Cell Sorter (FACS) was used to sort single *T. cruzi* cells directly into 96-wells microplates. Cells were then genotyped using two polymorphic genomic markers and four microsatellite loci. We validated this methodology by testing four *T. cruzi* populations: one control artificial mixture composed of two monoclonal populations – Silvio X10 cl1 (TcI) and Esmeraldo cl3 (TcII) – and three naturally occurring strains, one isolated from a vector (A316A R7) and two others derived from the first reported human case of Chagas disease. Using this innovative approach, we were able to successfully describe the whole complexity of these natural strains, revealing their multiclonal status. In addition, our results demonstrate that these *T. cruzi* populations are formed of more clones than originally expected. The method also permitted estimating of the proportion of each subpopulation of the tested strains. The single-cell genotyping approach allowed analysis of intrapopulation diversity at a level of detail not achieved previously, and may thus improve our comprehension of population structure and dynamics of *T. cruzi*. Finally, this methodology is capable to settle once and for all controversies on the issue of multiclonality.

## Author Summary

*Trypanosoma cruzi*, the cause of Chagas disease, is responsible for high levels of mortality and morbidity in many countries of Latin America. This species is very polymorphic and was recently classified into six discrete typing units (TcI-VI) that are associated with different geographical distribution, transmission cycles, and varying disease symptoms. Natural parasite populations are thought to have a complex structure since in endemic areas the same hosts may be simultaneously infected by different *T. cruzi* strains due to frequent exposure to insects as well as the variety of reservoirs and vectors in the sylvatic environment and the heterogeneous composition of the parasite populations. Most of the putative multiclonal populations have been suggested based on indirect evidence, or current conventional cloning methods that may favor some individuals' growth. In an effort to elucidate the complexity of *T. cruzi* isolates we adapted a methodology originally described for analyzing single spermatozoids, to sort single parasites. This approach allowed us to completely dissect the complexity of four strains and estimate the relative contribution of each subpopulation. Knowledge about complexity of *T. cruzi* strains is essential for determining the aspects involved in differential parasite tissue tropism, clinical manifestations of the disease, and drug resistance.

## Introduction

Chagas disease, an American protozoonosis caused by *Trypanosoma cruzi*, is characterized by various clinical manifestations ranging from asymptomatic to severe cardiac and/or digestive injuries. This complex ailment still afflicts 10 million people across Latin America and another 90 million are at risk of acquiring the disease [1]. A vaccine or specific treatment for large-scale public health interventions is still not available, so the main control strategy relies on prevention of transmission by controlling insect vectors and contamination by food or blood transfusion [2].

*T. cruzi* is a very polymorphic species, as extensively demonstrated by biological, biochemical, and molecular studies [3], and this certainly contributes to the pleomorphism of the symptoms and to the difficulty in controlling the disease [4]. *T. cruzi* was recently subdivided into six discrete taxonomic units (DTUs) named *T. cruzi* I to *T. cruzi* VI [5], of which at least four are involved with human pathology [6].

Indirect evidence suggests that part of *T. cruzi* populations is multiclonal. This is in accordance with the theoretical expectation, since patients in endemic areas are infected by multiple contacts with different triatomines and these, in turn, may feed on different infected individuals. Most of the putative multiclonal populations were suggested based on the identification of more than two alleles for different markers [7]–[9]. Whether these reported populations are really multiclonal remains controversial because we cannot exclude the possibility of aneuploidy [7], [8], [10], [11]. Thus, direct evidence for the multiclonality of sylvatic and domestic populations is lacking. Indeed, it is not possible to identify all the clones that constitute a given multiclonal population because current conventional cloning methods (micromanipulation, limiting dilution or cloning in blood-agar plates) may favor individuals over-represented in the original population and/or presenting higher growth rates [12]–[15].

To answer these questions we devised a new strategy for sorting single *T. cruzi* parasites, adapted from methods originally described for analyzing single spermatozoids [16]. Using this innovative approach the results presented herein may contribute to settling the debate regarding the multiclonality of three *T. cruzi* representative strains isolated from human and vector hosts. The methodology described here is able to completely dissect the complexity of strains and estimate the relative contribution of each subpopulation, and will therefore have important implications for the study of *T. cruzi* biology and disease.

## Methods

### *T. cruzi* populations

In this study we used four multiclonal *T. cruzi* populations: one artificially composed of Esmeraldo cl3 (a standard *T. cruzi* II strain) and Silvio X10 cl1 (a standard *T. cruzi* I strain), a naturally occurring putatively multiclonal vector strain called A316A R7, and two populations derived from a naturally occurring putatively multiclonal human strain named Be-78 1B and Be-78 25B. The A316A R7 strain was isolated from a *Triatoma sordida* circulating in northwestern Paraná state, Brazil [17], [18]. The Be-78 1B and Be-78 25B are two populations derived from a chronic chagasic outbred dog infected with Be-78, originally isolated from Berenice, the first patient of Carlos Chagas [19], harvested after 1 or 25 successive blood passages in mice [20].

### Preparation of *T. cruzi* cells for the FACS

About 1.5 mL (10^6^ cells) of *T. cruzi* epimastigotes cultured in Liver Infusion Tryptose (LIT) medium were transferred to a siliconed Vacutainer tube (Itupeva, Brazil). The culture was centrifuged at 30×g for 10 minutes at 4°C to separate intact cells from cellular debris. After this centrifugation, the culture was incubated for 10 minutes at room temperature to allow mobile epimastigotes to reach the surface. The collected cells were submitted to three washes with 1× PBS pH 7.4 at 4°C sterilized using 0.22 µM Millex GV filter (Millipore, MA, USA) and centrifuged at 1000×g for 10 minutes at 4°C. After the last wash the cells were suspended in 1 mL of 1× PBS pH 7.4 and fixed for 30 minutes using absolute ethanol maintained at −20°C. Finally, the fixed cells were stored at 4°C overnight, after which they were submitted to sorting on the FACS Vantage apparatus.

### Calibrating the FACS and sorting of *T. cruzi* cells

To calibrate the powerful single-cell sorter FACS Vantage to achieve single *T. cruzi* specimens, we used fluorescent beads as an indirect control because bead size is similar to the epimastigote form of the parasite [21]. Fluorescent beads (Spherotech Inc., IL, USA) measuring 10.0 to 14.0 µm in diameter were sorted in microscope slides according to the manufacturer's protocol (Becton Dickinson, CA, USA). We found that the counting of the sorted beads under fluorescence microscope always showed good correspondence with the number of beads programmed for each well. In wells programmed to contain one bead we found an occasional absence of beads, but never more than one bead.

To sort *T. cruzi* strains, small aliquots of the fixed cells were transferred to a Falcon tube (Becton Dickinson, CA, USA) for FACS counting. The solution containing the parasites was diluted in 1× PBS pH 7.4 until the FACS counting indicated approximately 100 events per second. Samples were gated forward scatter (FSC) versus side scatter (SSC) to select cell populations of interest (Figure S1). The autofluorescence pattern presented by *T. cruzi* allows cell sorting without staining with fluorescent dyes or reagents. Then, the Clone Cyt apparatus (Becton Dickinson, CA, USA) was used to sort single cells directly into 96-wells plate (Sorensen BioScience, UT, USA) containing 5 µL of 10% Triton X-100. Control wells were programmed to contain 0, 2, 5, or 10 sorted parasites from each *T. cruzi* population. After sorting, a drop of mineral oil was added to each well and the microplates were stored at −20°C until needed. Before the PCR assays, the single sorted *T. cruzi* was lysed by exposing the microplates to 80°C for 1 hour in a thermocycler.

### *T. cruzi* single cell genotyping

Cells were genotyped by using polymorphic markers to mitochondrial (Cytochrome Oxidase subunit II- COII) and nuclear (24Sα rDNA) genes and four microsatellite loci.

#### Polymorphism analysis of 24Sα rDNA gene

A hemi-nested PCR protocol was used for the 24Sα rDNA typing. Regular D75/D72 and 5′-fluorescein D71/D72 primer pairs (Table 1) were used for the first and second rounds, respectively. Both rounds of each PCR were performed in a total volume of 25 µL containing Buffer B (Promega, Madison, Wisconsin, USA), 3.5 mM MgCl_2_, 0.625 U *Taq* DNA polymerase (Promega), 200 µM of each dNTP, and 0.25 µM of each primer. The lysed single *T. cruzi* cells were used as DNA template for the first round and 10% of the amplified products obtained from this round were used for the second. The amplification cycles for both rounds consisted of an initial denaturation step at 94°C for 1 minute, followed by 30 cycles of 94°C, 60°C, and 72°C for 30 seconds each step, followed by final extension step at 72°C for 10 min [22]. After the second round, 5 µL of the PCR products were examined on silver stained 6% polyacrylamide gel. As PCR amplification controls we used 1 ng of DNA from *T. cruzi* populations belonging to *T. cruzi* I: Silvio X10 cl1 (rDNA type 2 – 110 bp); or *T. cruzi* II: Esmeraldo cl3 (rDNA type 1–125 bp) and *T. cruzi* V: SO3 cl5 (rDNA type 1/2 – 110 and 125 bp). To determinate the allele sizes, 1 to 3 µL of the PCR fluorescent products were analyzed on 6% denaturing polyacrylamide gel with an Automatic Laser Fluorescent (ALF) sequencer (GE Healthcare, Milwaukee, Wisconsin, USA) in comparison to fluorescent DNA fragments of 50–500 bp using Allelelocator software (GE Healthcare).

**Table 1:** Primers used for amplification of the *T. cruzi* 24Sα rDNA, COII, and microsatellite markers.

Primer	Sequence
**D71**	5′-FluoresceinAAGGTGCGTCGACAGTGTGG-3′
**D72**	5′-TTTTCAGAATGGCCGAACAGT-3′
**D75**	5′-CAGATCTTGGTTGGCGTAG-3′
**TcMit10**	5′-CCATATATTGTTGCATTATT-3′
**TcMit21**	5′-TTGTAATAGGAGTCATGTTT-3′
**DsMit-F**	5′-TGCATTACTCCTTTCTACAG-3′
**DsMit-R**	5′-AACTCGCTACATTGTCCATA-3′
**TcAAT8-forward**	5′-FluoresceinACCTCATCGGTGTGCATGTC-3′
**TcAAT8-reverse**	5′-TATTGTCGCCGTGCAATTTC-3′
**TcAAT8ex-forward**	5′-AGAGGCGCACAGTTGTATGC-3′
**TcAAT8ex-reverse**	5′-GACGCTTTATGTTGAATTCA-3′
**TcTAC15-forward**	5′-FluoresceinGAATTTCCCCATTTCCAAGC-3′
**TcTAC15-reverse**	5′-CGATGAGCAACAATCGCTTC-3′
**TcTAC15ex-forward**	5′-GGATATTTGTTACTGCTGGC-3′
**TcTAC15ex-reverse**	5′-CGGACATATCCCTCTAGTCG-3′
**TcTAT20-forward**	5′-FluoresceinGATCCTTGAGCAGCCACCAA-3′
**TcTAT20-reverse**	5′-CAAATTCCCAACGCAGCAGC-3′
**TcTAT20ex-forward**	5′-AGGCTGATCCTTGAGCAGCC-3′
**TcTAT20ex-reverse**	5′-CGGCGGTCTTCTTTTGTCTC-3′
**TcAAAT6-forward**	5′-FluoresceinGCCGTGTCCTAAAGAGCAAG-3′
**TcAAAT6-reverse**	5′-GGTTTTAGGGCCTTTAGGTG-3′
**TcAAAT6ex-forward**	5′-ACGCACTCTCTTTGTTAACAG-3′
**TcAAAT6ex-reverse**	5′-CACATACACATTCCAATGGTT-3′

#### Polymorphism analysis of COII gene

COII typing was conducted using a full nested PCR protocol as described previously [23], [24]. DsMit-F/DsMit-R and TcMit10/TcMit21 primer pairs (Table 1) were used for the first and the second PCR rounds, respectively. Each reaction was performed in a total volume of 25 µL containing 2.5 µL of Buffer IB (Phoneutria, Minas Gerais, Brazil), 1 U *Taq* DNA polymerase (Promega), 250 µM of each dNTP, and 0.3 µM of each primer. The lysed single *T. cruzi* cells were used as DNA template for the first round and 10% of the amplified products obtained from this round were used for the second round. The cycles of PCR amplification consisted of an initial denaturation at 94°C for 5 minutes, followed by 40 cycles at 94°C for 45 seconds, 48°C for 45 seconds, and 72°C for 1 minute, followed by a final extension step at 72°C for 5 min. The amplified products were resolved on 2% agarose gel electrophoresis. Each positive PCR reaction (10 µL) was submitted to digestion with *Alu*I enzyme (Promega), according to the manufacturer's instructions. Restriction Fragment Length Polymorphism (RFLP) of this gene allows the differentiation of *T. cruzi* I, II, and III-VI major lineages when resolved on silver stained 6% polyacrylamide gel. As RFLP-COII standards patterns we used 1 ng of DNA from *T. cruzi* populations belonging to *T. cruzi* I: Sílvio X10 cl1 (mitochondrial haplotype A – 30, 81, and 264 bp); *T. cruzi* II: Esmeraldo cl3 (mitochondrial haplotype C – 81, 82, and 212 bp); and *T. cruzi* V: SO3 cl5 (haplotype B – 81 and 294 bp).

#### Polymorphism analysis of microsatellite loci

A multiplex PCR system based on the full nested PCR strategy was designed for the microsatellite analyses. This system comprised two amplification rounds. In the first round we had a multiplex PCR containing only the external primers for all four microsatellite loci identified in diploid regions of CL Brener genome (TcTAC15, TcAAT8, TcTAT20, and TcAAAT6) (Table 1). For the second round, four different reactions were carried out using the internal primers for each locus individually. Both PCR rounds were performed in a total volume of 25 µL containing Buffer B (Promega), 2.5 mM MgCl_2_, 1 U *Taq* DNA polymerase (Promega), 300 µM of each dNTP, and 0.3 µM of each primer. Lysed single *T. cruzi* cells were used as DNA template for the first round and 10% of the amplified products obtained from this round were used for the second. Amplification reactions were performed using the step-down protocol described by Valadares et al. (2008). To determinate allele sizes, 1 to 3 µL of the PCR fluorescent products were analyzed on 6% denaturing polyacrylamide gel with an ALF sequencer (GE Healthcare).

#### Combining 24Sα rDNA and microsatellite analyses

To investigate whether there was a correspondence between the microsatellite and rDNA profiles of the single sorted *T. cruzi*, we used a duplex PCR involving the simultaneous amplification of the 24Sα rDNA and TcTAT20 markers. The reactions were conducted as specified above for the microsatellite analyses in a two-round PCR. For the first round we used a mixture of the external primers for 24Sα rDNA and TcTAT20, and in the second round we used the internal primers for both markers but in two separate reactions.

## Results

Conventional cloning methods have showed the existence of some natural multiclonal *T. cruzi* populations. However, a widespread understanding of the multiclonal scenario among *T. cruzi* is not possible due to the limitations of current cloning methods. We therefore wanted to devise a method that would allow us to investigate the multiclonal status of any *T. cruzi* strain and simultaneously characterize individually all the clones that compose the strains.

### Single sorting of *T. cruzi* parasites using FACS Vantage

**Figure 1:**
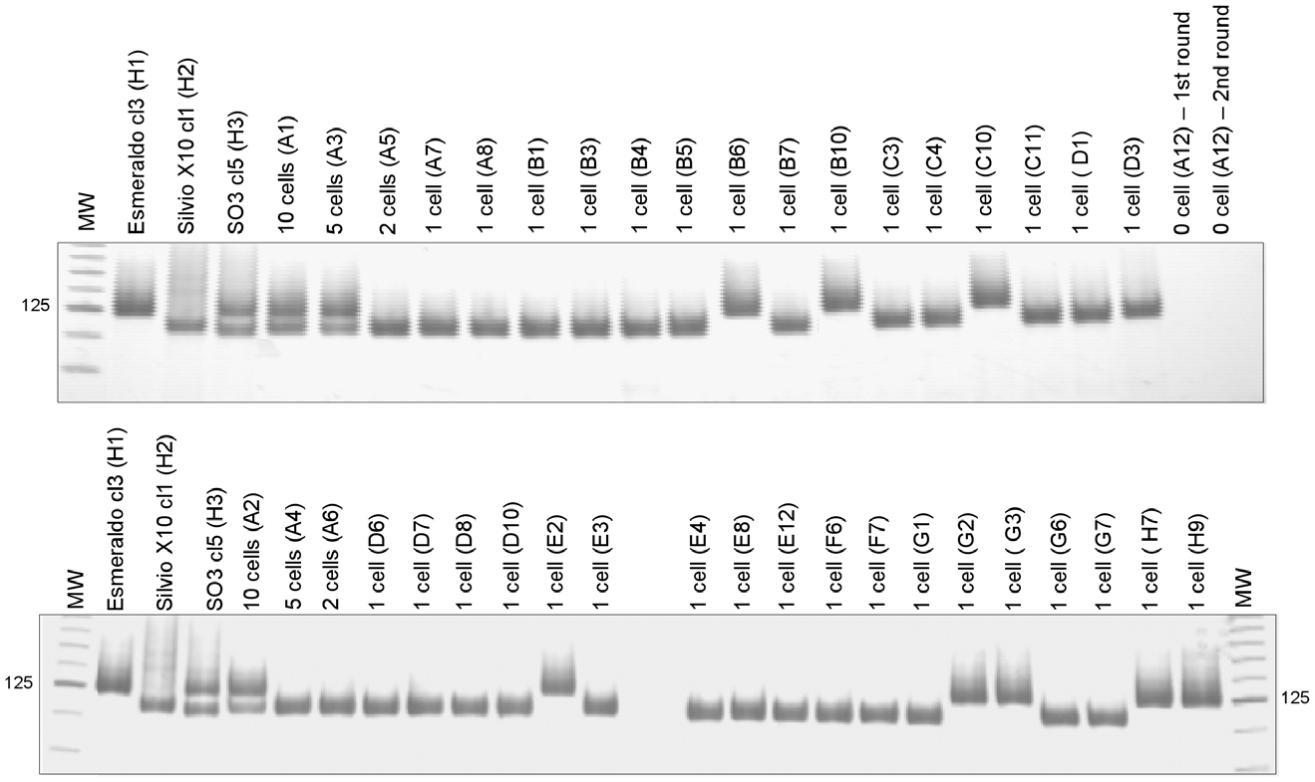
24Sα rDNA profiles of single parasites from the SilvioX10 cl1 and Esmeraldo cl3 artificial mixture. DNA from Esmeraldo cl3 (125 bp), Silvio X10 cl1 (110 bp), and SO3 cl5 (110/125) were used as controls for rDNA type 1 (*T. cruzi* II), rDNA type 2 (*T. cruzi* I), and rDNA type 1/2 (*T. cruzi* V), respectively. Corresponding microplate wells of the positive amplifications are also indicated. Control wells were programmed to contain 0, 2, 5, or 10 parasites. MW: Molecular weight of 25 bp (Invitrogen, USA).

To evaluate whether the previously used bead-calibrated method could be successfully used to separate single *T. cruzi* parasites, we initially sorted an artificial mixture of two monoclonal populations: Silvio X10 cl1 (*T. cruzi* I) and Esmeraldo cl3 (*T. cruzi* II). Next, we confirmed separation by genotyping the 24Sα rRNA gene of the single sorted parasites and the controls present in each well (Figure 1). In the control wells A1, A2, A3, and A4 programmed to contain 10 or 5 cells, we detected the expected amplicons of 110 and 125 bp, demonstrating that the two types of cells used in the artificial mixture were present. From the 83 wells programmed to contain the sorted single parasites, 40% (33 wells) showed the amplification of only the 125 bp or the 110 bp amplicon, therefore confirming single cell sorting. This efficiency is approximately the double that obtained with a previously published protocol [25]. Moreover, none of the wells programmed to contain a single cell showed the amplification of both fragments. These results indicate that the FACS Vantage sorting method we modified could be used efficiently to separate single *T. cruzi* parasites.

### Single parasite sorting of naturally occurring vector populations

**Figure 2:**
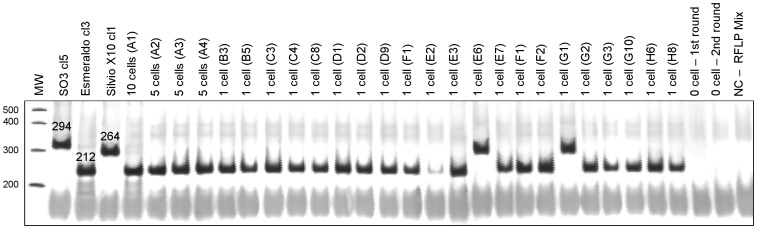
PCR-RFLP profiles of the COII gene from the A316A R7 single sorted parasites. After digestion with *Alu*I the COII amplicons generate three RFLP patterns for *T. cruzi*: the presence of a fragment of 264 bp characterizes a mitochondria haplotype A, associated with *T. cruzi* I (e.g., Silvio X10 cl1); the presence of a fragment of 212 bp characterizes a mitochondria haplotype C, associated with *T. cruzi* II (e.g., Esmeraldo cl3), and the presence of a fragment of 294 bp characterizes a mitochondria haplotype B associated with *T. cruzi* III-VI DTUs (e.g., S03 cl5). Only wells that resulted in positive amplification are shown. Control wells were programmed to contain 0, 2, 5, or 10 parasites. MW: Molecular weight of 25 bp (Invitrogen, USA); NC – RFLP mix: negative control containing only the reaction mix.

**Figure 3:**
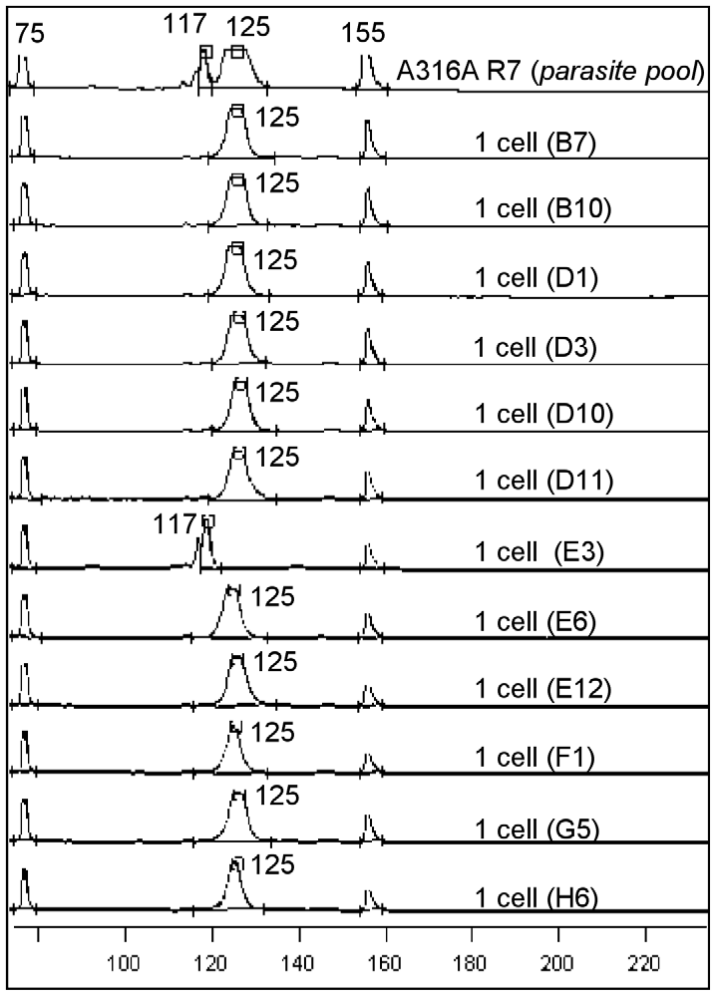
Electrofluorogram presenting the profiles obtained for 24Sα rDNA on A316A R7 single sorted parasites. Numbers at the peaks refer to the size of the amplicons in base pairs. The peaks presenting values of 75 and 155 bp refer to internal markers used for alignment in the Allelelocator software. The wells containing single cells that showed positive amplification are also indicated. Control wells presenting DNA of the original strain pool of parasites or 10 sorted parasites were also included.

**Figure 4:**
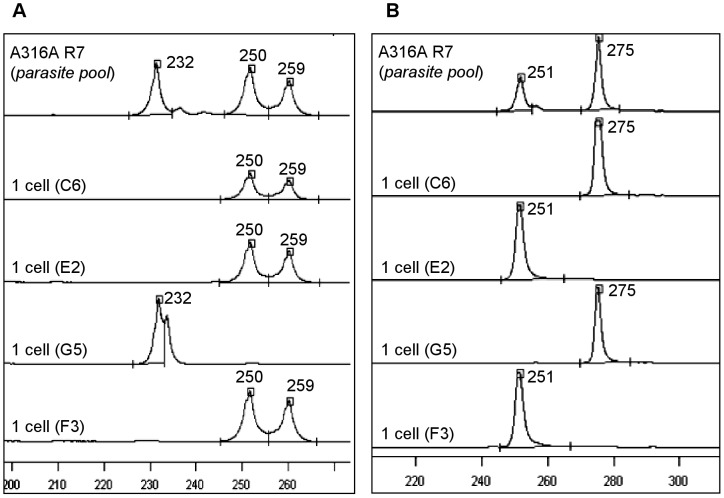
Electrofluorogram of TcAAT8 (A) and TcAAAT6 (B) loci for A316A R7 before and after sorting. Numbers at the peaks refer to the size of the amplicons in base pairs. The peaks presenting values of 75, 210, and 320 bp refer to internal markers used for alignment in the Allelelocator software. The wells containing single cells that showed positive amplification are also indicated. Control wells presenting DNA of the original strain pool of parasites were also included.

The A316A R7 strain was isolated from a triatomine bug in Paraná, southern Brazil. Because this strain presented more than two alleles for some of the DNA markers used during its genotyping, it was suggested that it was constituted of a mixed population [17]. However, considerable controversy persisted on whether the multiclonal status of this and other *T. cruzi* strains is real [7], [8], [26]–[28] or just reflects aneuploidy for the analyzed loci [10], [11]. We therefore used the single *T. cruzi* sorting and genotyping approach proposed here to address this question. To this end, we sorted single parasites from the A316A R7 strain and genotyped them by using three sets of markers: COII, 24Sα rDNA, and microsatellites (Figures 2, 3, 4).

#### COII haplotypes

The COII profiles obtained for the single sorted parasites and controls are shown in Figure 2. On average 25% of the wells programmed to contain a single cell were positive for this marker. We detected two different RFLP profiles, demonstrating the presence of two subpopulations within the A316A R7 strain: one showing the fragment of 212 bp, which is characteristic of the mitochondrial COII haplotype C and shared by strains belonging to *T. cruzi* II, and another showing the fragment of 264 bp, which is characteristic of the mitochondrial COII haplotype A, and shared by strains belonging to *T. cruzi* I. We also estimated the relative proportion of each subpopulation within the parental A316A R7 strain and found that the *T. cruzi* II parasites were more frequent (approximately 90%) than the *T. cruzi* I (approximately 10%).

#### 24Sα rDNA profiles

Figure 3 shows that 42% of wells programmed to contain a single sorted *T. cruzi* were positive for this marker. We detected two different profiles: one presenting 125 bp, compatible with strains belonging to *T. cruzi* II lineage, and another presenting a fragment of 117 bp previously demonstrated for some non-typical *T. cruzi* I strains [29] (Figure 3). Similar to the data obtained by mitochondrial COII haplotype analysis, we also observed that the *T. cruzi* II population predominates in the original strain (approximately 85%).

#### Microsatellite typing

Representative results obtained from the microsatellite analyses of the single sorted *T. cruzi* and the controls wells are shown on Figure 4. On average 34% of the wells programmed to contain a single cell were positive for the microsatellites, varying from 23 to 41% depending on the experiment and the evaluated locus. The compiled data derived from all analyzed loci are presented in Table 2. When we analyzed pools of parasites obtained from the A316A R7 strain we detected three alleles for the TcAAT8 locus (232, 250, and 259 bp), which could be indicative of multiclonality or aneuploidy for this strain. However, the PCR analysis of the same locus performed with the single sorted cells detected two distinct subpopulations: one presenting 232 bp alleles (homozygote profile) and another showing alleles of 250 and 259 bp (heterozygote profile). Similar results were obtained for the other analyzed loci (TcTAT20, TcTAC15, and TcAAAT6). Although the analyses performed with these other loci identified profiles with only two alleles in the original A316A R7 strain, PCR using the single sorted cells allowed us to detect two distinct subpopulations. Indeed, two different but homozygous profiles for the TcTAC15 (96/96 or 99/99 bp) and the TcAAAT6 (251/251 or 275/275 bp) loci were detected. Moreover, the TcTAT20 locus revealed also the presence of two subpopulations: one heterozygous, presenting the 190/238 bp alleles, and another homozygous with the 181/181pb, which is curious given that this last allele was not detected in the original strain.

**Table 2:** Allele sizes for each microsatellite locus amplified from A316A R7 single sorted cells.

Sample/Loci	TcTAC15	TcTAT20	TcAAT8	TcAAAT6
A316A R7 (strain)	99/99	190/238	232/250/259	251/275
1 cell	99/99	190/-	250/259	n.a
1 cell	99/99	190/238	-/259	n.a
1 cell	99/99	190/-	n.a	275/275
1 cell	99/99	190/238	250/259	275/275
1 cell	96/96	181/181	n.a	251/251
1 cell	99/99	190/-	n.a	275/275
1 cell	99/99	190/238	250/259	275/275
1 cell	99/99	190/-	250/259	275/275
1 cell	99/99	190/-	250/259	275/275
1 cell	99/99	190/-	250/-	275/275
1 cell	96/96	181/181	232/232	251/251

Allele sizes are presented in base pairs.

(-), Amplification failure probably due to allele drop-out effect; n.a, not amplified.

Perfect correspondence among the alleles found for each microsatellite locus confirmed the existence of two subpopulations within the A316A R7 strain presenting completely different genotypes: one characterized by the 99/99, 190/238, 250/259, and 275/275 bp alleles and another characterized by the 96/96, 181/181, 232/232, and 251/251 bp alleles for the TcTAC15, TcTAT20, TcAAT8, and TcAAAT6 microsatellite loci, respectively (Table 2). The proportion of wells presenting each of these two subpopulations was estimated as 90% and 10% respectively, a result consistently found from experiments performed using different microplates.

#### Correlating the microsatellite analyses with the *T. cruzi* DTUs

To determine the *T. cruzi* DTUs for the two subpopulations identified by the microsatellite analyses, we used a duplex PCR strategy designed to simultaneously amplify the TcTAT20 and the 24Sα rDNA markers (Table 3). Our results demonstrated that the subpopulations presenting the 181/181 and 190/238 bp alleles for the TcTAT20 locus corresponded, respectively, to those presenting the 24Sα rDNA of 117 bp and associated with the *T. cruzi* I, and the 24Sα rDNA of 125 bp related to the *T. cruzi* II, both present in the original A316A R7 strain (Table 3).

**Table 3:** Allele sizes of TcTAT20 microsatellite and 24Sα rDNA gene amplified from A316A R7 single cells.

Sample/Loci	TcTAT20	24Sα rDNA	*T. cruzi* DTU
A316A R7 (strain)	190/238	117/125	*T. cruzi* I+*T. cruzi* II
1 cell	190/238	125	*T. cruzi* II
1 cell	190/-	125	*T. cruzi* II
1 cell	190/238	125	*T. cruzi* II
1 cell	181/181	117	*T. cruzi* I

Allele sizes are presented in base pairs.

(-), Amplification failure probably due to allele drop-out effect. *T. cruzi* DTUs according to Zingales *et al.*, 2009.

In this table are shown only those wells that presented positive amplification for both markers.

#### Single parasite sorting of naturally occurring human populations

Be-62 and Be-78 are two *T. cruzi* strains isolated respectively in 1962 and 1978 from Berenice, the first reported human case of Chagas disease [30]. Although isolated from the same patient, these two strains displayed several biological and molecular differences [19], [31]–[33]. However, because only two alleles were detected for the molecular markers used in their characterization, both strains were initially supposed to be monoclonal [34]. Even so, after being inoculated in dogs and mice and re-isolated, major changes in RAPD and isoenzyme profiles were observed in the Be-78 profile, raising the question whether this strain could be indeed a multiclonal population [20]. To answer this query we used the methodology proposed here for sorting Be-78 1B and Be-78 25B, two of the isolates from the Be-78 strain obtained after passages in dog and then in mice. The microsatellite profiles obtained were compared with those observed for the original Be-62 and Be-78 strains and these data were summarized in Table 4. Surprisingly, three different subpopulations were identified: one characterized by the 99/99, 196/205, 262/268, and 271/275 bp alleles and equivalent to the original profile of Be-78; another one characterized by the 99/99, 190/208, 253/262, and 259/275 bp alleles and compatible with the original profile of Be-62; and a third one characterized by the 138/141, 181/181, 292/292, and 263/263 bp alleles for the TcTAC15, TcTAT20, TcAAT8, and TcAAAT6 microsatellite loci, respectively.

**Table 4:** Allele sizes for each microsatellite amplified from Be-78 1B and Be-78 25B single sorted cells.

Sample/Loci	TcTAC15	TcTAT20	TcAAT8	TcAAAT6
Be-62 (Strain)	99/99	190/208	253/262	259/275
Be-78 (Strain)	99/99	196/205	262/268	271/275
Be-78 1B (Isolate)	99/138/141	190/196/205	253/253	259/275
1 cell Be-78 1B	99/99	n.a	262/268	271/275
1 cell Be-78 1B	138/141	n.a	292/292	263/263
1 cell Be-78 1B	99/99	a.a	253/262	259/275
1 cell Be-78 1B	99/99	n.a	262/268	271/275
1 cell Be-78 1B	138/141	n.a	292/292	263/263
Be-78 25B (Isolate)	99/138/141	181/190/208	253/262/268/292	259/275
1 cell Be-78 25B	99/99	190/208	253/-	259/-
1 cell Be-78 25B	99/99	190/208	253/262	259/275
1 cell Be-78 25B	99/99	190/208	253/262	-/275
1 cell Be-78 25B	138/141	181/181	292/292	263/263
1 cell Be-78 25B	99/99	190/208	253/-	259/275
1 cell Be-78 25B	138/141	181/181	n.a	263/263
1 cell Be-78 25B	138/141	181/181	292/292	263/263
1 cell Be-78 25B	99/99	190/208	253/262	259/275
1 cell Be-78 25B	99/99	190/208	253/262	-/275
1 cell Be-78 25B	99/99	190/208	253/-	259/275
1 cell Be-78 25B	138/141	181/181	292/292	263/263

Allele sizes are presented in base pairs.

(-), Amplification failure probably due to allele drop-out effect; n.a, not amplified or not analyzed.

In this table are shown the alleles from wells derived from different experiments that presented positive amplification for at least three microsatellite loci.

Taken together, our results confirm that the devised strategy was able to sort single parasites and reveal that the analyzed naturally occurring vector and human strains are indeed multiclonal and not aneuploid *T. cruzi* populations.

## Discussion

To bring further light to the debate regarding the multiclonal status of *T. cruzi* strains, an innovative approach was successfully adapted from a method originally described to genotype single sorted spermatozoids, to separate single parasites. The evidence described herein reveals that multiclonality holds true for two representative *T. cruzi* human and vector strains. The approach we developed employed the FACS Vantage sorting method, which allowed us to estimate the relative contribution of each parasite subpopulation within the strains analyzed.

The efficiency of the approach was initially established by sorting and genotyping single parasites from an artificially composed multiclonal population and not only all the individuals present in the original mixture could be identified, but also the real proportion of each population could be assigned.

The devised method was then used to scrutinize three naturally occurring putative multiclonal populations originally isolated from a vector (A316A R7) and two derived from a patient (Be-78 1B and Be-78 25B). A long-held debate was whether *T. cruzi* strains presenting more than two alleles for some polymorphic markers were really multiclonal or aneuploid populations [7], [9], [10].

Previous work suggested that the A316A R7 strain was a mixture of distinct populations [18], [29]. Our new results involving the microsatellite analysis of single sorted parasites confirmed the presence of different subpopulations within this strain, ruling out aneuploidy as a possible explanation for the complex genetic patterns presented by this population.

Interesting results were observed with the human *T. cruzi* strain. Great controversy remains on whether or not Berenice, the first patient of Carlos Chagas, has been infected with a multiclonal *T. cruzi*. This was due to the fact that two different but apparently monoclonal populations (Be-62 and Be-78) were isolated from her [34]. The observation that Be-78 has changed its molecular and biochemical characteristics after passages in dogs and mice strengthened this possibility, suggesting that the passage in different hosts could have grown previously undetectable subpopulations within the original strain. Here we showed that Be-78 1B and Be-78 25B (two of the *T. cruzi* populations derived from a dog infected with Be-78 after 1 or 25 successive passages in mice) were constituted by three subpopulations: one compatible with Be62, another equivalent to the Be78, and a third one not identified before. Therefore, the complexity of this *T. cruzi* strain is even greater than originally expected.

We also detected alleles not previously reported for the analyzed strains. The probable reason they were not found before is that when alleles of less representative subpopulations are lower than 15% in the DNA artificial mixture, they cannot be detected by conventional PCR (our unpublished data).

Another remarkable result was observed regarding the TcAAAT6 profiles of the single sorted parasites. The analysis of the vector strain showed the presence of two alleles of 251 and 275 bp, initially interpreted as a typical heterozygote genotype. However, the analysis of the single parasites using this locus showed the presence of two different subpopulations both presenting homozygote profiles. This finding emphasizes the advantage of genotyping single cells instead of pools of parasites, because conventional PCR cannot distinguish between the presence of a homogeneous population presenting two different alleles, and a heterogeneous population constituted by two different and homozygous individuals. Interestingly, by using the methodology presented here we successfully identified the subpopulations presented in a multiclonal strain as well as estimate the proportion of each subpopulation.

A drawback inherent to use of a single cell is failure of amplification of one of the two alleles of some loci, a phenomenon known as allele drop-out (ADO). Due to its characteristics, ADO can be misleading as false homozygosity, as one of the alleles in heterozygote loci is undetectable [35]. Although we observed ADO in some of our analyses, it did not interfere with the single-cell genotyping because we analyzed simultaneously 96 plated cells and a variety of loci. Contrasting to real homozygosis, ADO is a random phenomenon and it does not occur in the same way in all wells and loci. Thus, the more plates and therefore the more single cells analyzed, more reliable the results will be, a possibility easily achievable by the cell sorting method devised.

In summary, by using the strategy proposed here for sorting and genotyping *T. cruzi* single cells we successfully identified the complexity of representative human and vector *T. cruzi* strains and estimated the relative contribution of each subpopulation. These results have important implications in settling the debate regarding multiclonality versus aneuploidy in these representative strains. Moreover, our results indicate that the complexity of *T. cruzi* strains may be even greater than initially expected.

It is worth noting that the methodology described herein has the potential to close the discussion concerning multiclonality. Knowledge about complexity of *T. cruzi* strains is essential for determining the aspects involved in differential parasite tissue tropism, clinical manifestations of the disease, and drug resistance. The single-cell genotyping approach allowed the analysis of the intrapopulation diversity to a degree not achieved previously by conventional methods, and may represent a powerful and new tool for the understanding of the population structure and dynamics of *T. cruzi*.

## Supporting Information

Figure S1 **Dot plot captured by FACS showing the autofluorescence pattern and the gated** ***T. cruzi*** **cells.**
(TIFF)

## References

[1] Coura JR, Dias JC (2009). Epidemiology, control and surveillance of Chagas disease: 100 years after its discovery.. Mem Inst Oswaldo Cruz.

[2] Dias JC, Silveira AC, Schofield CJ (2002). The impact of Chagas disease control in Latin America: a review.. Mem Inst Oswaldo Cruz.

[3] Macedo AM, Pena SD (1998). Genetic Variability of Trypanosoma cruzi:Implications for the Pathogenesis of Chagas Disease.. Parasitol Today.

[4] Vago AR, Andrade LO, Leite AA, d'Avila Reis D, Macedo AM (2000). Genetic characterization of Trypanosoma cruzi directly from tissues of patients with chronic Chagas disease: differential distribution of genetic types into diverse organs.. Am J Pathol.

[5] Zingales B, Andrade SG, Briones MR, Campbell DA, Chiari E (2009). A new consensus for Trypanosoma cruzi intraspecific nomenclature: second revision meeting recommends TcI to TcVI.. Mem Inst Oswaldo Cruz.

[6] Miles MA, Llewellyn MS, Lewis MD, Yeo M, Baleela R (2009). The molecular epidemiology and phylogeography of Trypanosoma cruzi and parallel research on Leishmania: looking back and to the future.. Parasitology.

[7] Oliveira RP, Broude NE, Macedo AM, Cantor CR, Smith CL (1998). Probing the genetic population structure of Trypanosoma cruzi with polymorphic microsatellites.. Proc Natl Acad Sci U S A.

[8] Valadares HM, Pimenta JR, de Freitas JM, Duffy T, Bartholomeu DC (2008). Genetic profiling of Trypanosoma cruzi directly in infected tissues using nested PCR of polymorphic microsatellites.. Int J Parasitol.

[9] Macedo AM, Machado CR, Oliveira RP, Pena SD (2004). Trypanosoma cruzi: genetic structure of populations and relevance of genetic variability to the pathogenesis of chagas disease.. Mem Inst Oswaldo Cruz.

[10] Gaunt MW, Yeo M, Frame IA, Stothard JR, Carrasco HJ (2003). Mechanism of genetic exchange in American trypanosomes.. Nature.

[11] Lewis MD, Llewellyn MS, Gaunt MW, Yeo M, Carrasco HJ (2009). Flow cytometric analysis and microsatellite genotyping reveal extensive DNA content variation in Trypanosoma cruzi populations and expose contrasts between natural and experimental hybrids.. Int J Parasitol.

[12] Dvorak JA, Hall TE, Crane MS, Engel JC, McDaniel JP (1982). Trypanosoma cruzi: flow cytometric analysis. I. Analysis of total DNA/organism by means of mithramycin-induced fluorescence.. J Protozool.

[13] Engel JC, Dvorak JA, Segura EL, Crane MS (1982). Trypanosoma cruzi: biological characterization of 19 clones derived from two chronic chagasic patients. I. Growth kinetics in liquid medium.. J Protozool.

[14] Postan M, McDaniel JP, Dvorak JA (1984). Studies of Trypanosoma cruzi clones in inbred mice. II. Course of infection of C57BL/6 mice with single-cell-isolated stocks.. Am J Trop Med Hyg.

[15] Pinto AS, de Lana M, Bastrenta B, Barnabe C, Quesney V (1998). Compared vectorial transmissibility of pure and mixed clonal genotypes of Trypanosoma cruzi in Triatoma infestans.. Parasitol Res.

[16] Li  HCX, Arnheim N, Abelson JNSM (1991). Analysis of DNA sequence variation in single cells..

[17] Spitzner FL, de Freitas JM, Macedo AM, Toledo MJO, Araújo SM (2007). Trypanosoma cruzi - triatomine associations and the presence of mixed infections in single triatomine bugs in Paraná state, Brazil.. Acta Parasitologica.

[18] Abolis NG, Araujo SM, Toledo MJ, Fernandez MA, Gomes ML (2011). Trypanosoma cruzi I-III in southern Brazil causing individual and mixed infections in humans, sylvatic reservoirs and triatomines.. Acta Trop.

[19] Lana M, Chiari CA (1986). [Comparative biological characterization of Berenice and Berenice-78 strains of Trypanosoma cruzi isolated from the same patient at different times].. Mem Inst Oswaldo Cruz.

[20] Veloso VM, Romanha AJ, Lana M, Murta SM, Carneiro CM (2005). Influence of the long-term Trypanosoma cruzi infection in vertebrate host on the genetic and biological diversity of the parasite.. Parasitol Res.

[21] Santelo FH, Dost CK, Albuquerque S (1998). Morphometric characterization of a strain of Trypanosoma cruzi.. Mem Inst Oswaldo Cruz.

[22] Souto RP, Zingales B (1993). Sensitive detection and strain classification of Trypanosoma cruzi by amplification of a ribosomal RNA sequence.. Mol Biochem Parasitol.

[23] de Freitas JM, Augusto-Pinto L, Pimenta JR, Bastos-Rodrigues L, Goncalves VF (2006). Ancestral genomes, sex, and the population structure of Trypanosoma cruzi.. PLoS Pathog.

[24] Carranza JC, Valadares HM, D'Avila DA, Baptista RP, Moreno M (2009). Trypanosoma cruzi maxicircle heterogeneity in Chagas disease patients from Brazil.. Int J Parasitol.

[25] Macedo AM, Pimenta JR, Aguiar RS, Melo AI, Chiari E (2001). Usefulness of microsatellite typing in population genetic studies of Trypanosoma cruzi.. Mem Inst Oswaldo Cruz.

[26] Tibayrenc M, Ward P, Moya A, Ayala FJ (1986). Natural populations of Trypanosoma cruzi, the agent of Chagas disease, have a complex multiclonal structure.. Proc Natl Acad Sci U S A.

[27] Carneiro M, Chiari E, Goncalves AM, da Silva Pereira AA, Morel CM (1990). Changes in the isoenzyme and kinetoplast DNA patterns of Trypanosoma cruzi strains induced by maintenance in mice.. Acta Trop.

[28] Macedo AM, Martins MS, Chiari E, Pena SD (1992). DNA fingerprinting of Trypanosoma cruzi: a new tool for characterization of strains and clones.. Mol Biochem Parasitol.

[29] Spitzner FL, Freitas JM, Macedo AM, Toledo MJO, Araújo SM (2007). Trypanosoma cruzi - triatomine associations and the presence of mixed infections in single triatomine bugs in Paraná state, Brazil.. Acta Parasitologica.

[30] Chagas C (1909). Nova tripanozomiase humana. Estudos sobre a morfologia e o ciclo evolutivo do Schizotrypanum cruzi n. gen., sp., agente etiológico de nova entidade mórbida do homem.. Mem Inst Oswaldo Cruz.

[31] de Lana M, Chiari CA, Chiari E, Morel CM, Goncalves AM (1996). Characterization of two isolates of Trypanosoma cruzi obtained from the patient Berenice, the first human case of Chagas' disease described by Carlos Chagas in 1909.. Parasitol Res.

[32] de Lana M, Chiari E, Tafuri WL (1992). Experimental Chagas' disease in dogs.. Mem Inst Oswaldo Cruz.

[33] Araujo FM, Bahia MT, Magalhaes NM, Martins-Filho OA, Veloso VM (2002). Follow-up of experimental chronic Chagas' disease in dogs: use of polymerase chain reaction (PCR) compared with parasitological and serological methods.. Acta Trop.

[34] Cruz RE, Macedo AM, Barnabe C, Freitas JM, Chiari E (2006). Further genetic characterization of the two Trypanosoma cruzi Berenice strains (Be-62 and Be-78) isolated from the first human case of Chagas disease (Chagas, 1909).. Acta Trop.

[35] Piyamongkol W, Bermudez MG, Harper JC, Wells D (2003). Detailed investigation of factors influencing amplification efficiency and allele drop-out in single cell PCR: implications for preimplantation genetic diagnosis.. Mol Hum Reprod.

