# Supplementary figures and images for: Unequivocal Identification of Subpopulations in Putative Multiclonal Trypanosoma cruzi Strains by FACs Single Cell Sorting and Genotyping
Source: PLoS Negl Trop Dis. 2012 Jul 10;6(7):e1722. doi: 10.1371/journal.pntd.0001722 (PMC3393670; doi:10.1371/journal.pntd.0001722)

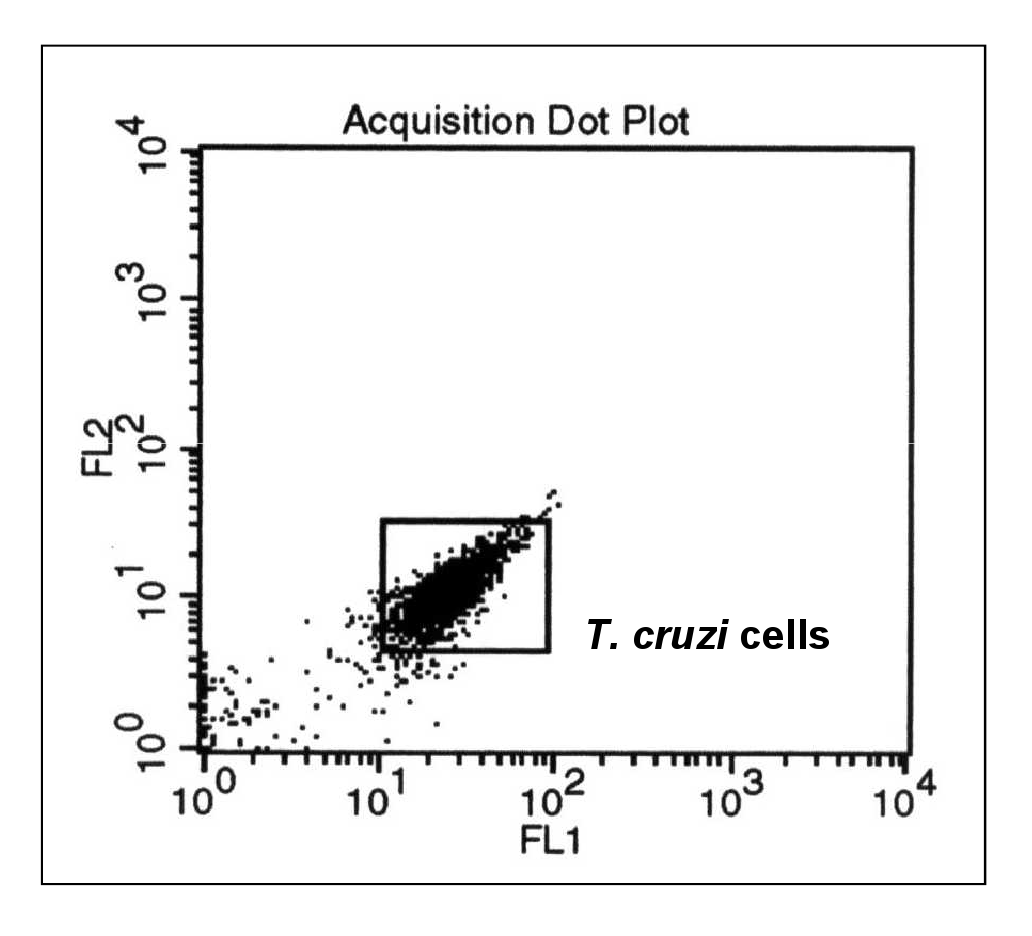

Supplement: Figure S1 — Dot plot captured by FACS showing the autofluorescence pattern and the gated T. cruzi cells. (TIFF) [file pntd.0001722.s001.tiff]
